# Outcomes and Complications of Scleral-Fixated Intraocular Lens Combined with Ahmed Tube Surgery

**DOI:** 10.1155/2018/9824035

**Published:** 2018-03-25

**Authors:** Nikhel Sachdev, Maria Cecilia Aquino, Seng Chee Loon, Yiong Huak Chan, Paul Chew, Victor Koh

**Affiliations:** ^1^Department of Ophthalmology, National University Hospital, Singapore 119228; ^2^Department of Biostatistics, Yong Loo Lin School of Medicine, Singapore 119228

## Abstract

**Background:**

To evaluate the outcome and complications of transscleral suture-fixated posterior chamber intraocular lens (PCIOL) implantation combined with Ahmed glaucoma valve (AGV) surgery in Asian eyes.

**Design:**

This was a retrospective study that included 22 eyes from 22 participants. The surgeries were performed at Singapore's National University Hospital. Participants underwent an Ahmed tube surgery, together with transscleral suture-fixated posterior chamber intraocular lens.

**Main Outcome Measures:**

Complete success was defined as 6 ≤ intraocular pressure (IOP) ≤ 21 mmHg without medications at the last follow-up visit, with no reoperation required and no progression to no perception of light (NPL).

**Results:**

At the last follow-up, there was a significant reduction in mean IOP (22.4 ± 6.5 mmHg versus 13.9 ± 3.9 mmHg; *p* < 0.001) and mean number of intraocular pressure-lowering medications (2.45 ± 1.30 versus 0.05 ± 0.21; *p* < 0.001). There was no significant change in visual acuity [1.43 ± 1.21 (LogMAR) versus 1.09 ± 1.31 (*p* = 0.204)]. Sixteen eyes (72.7%) achieved complete success. The 3 commonest complications were bullous keratopathy, choroidal detachment, and displacement of intraocular lens.

**Conclusion and Relevance:**

This technique showed good success for intraocular pressure control and vision preservation. Postoperative complications were relatively common although most were self-limiting. Patients at increased risk of trabeculectomy failure may be suitable for this procedure.

## 1. Introduction

Compared to anterior chamber lens, posterior chamber intraocular lens (IOL) implantation has several advantages including lower risk of corneal endothelial damage, superior visual outcomes, and less damage to anterior chamber angle structures [1, 2]. The last consideration is relevant in glaucoma patients in whom the trabecular meshwork function is already compromised [3]. Various techniques for the implantation of a posterior chamber IOL (PCIOL) in the absence of capsular support have been described, one of which is transscleral suture fixation [4]. When uncontrolled glaucoma coexists with a subluxed cataract or IOL, then a combined surgical approach in the same setting may be required [5]. This includes ocular trauma [6], congenital cataracts [7], connective tissue disease [6], and uveitic glaucoma [8].

Previous studies explored the option of transscleral suture-fixated PCIOL implantation combined with a trabeculectomy and found that this technique resulted in good intraocular pressure (IOP) control as well as improvement in visual acuity [3, 9]. To our knowledge, there is no data on the outcomes and safety profile of transscleral suture-fixated PCIOL combined with Ahmed glaucoma valve (AGV) implantation.

In the tube versus trabeculectomy (TVT) study, Gedde [10] compared the safety and efficacy of tube shunt surgery to trabeculectomy with mitomycin C, in the eyes that had prior intraocular surgery. The trabeculectomy group had significantly lower mean IOP than the tube group at all follow-up visits during the first 3 months (at 3 months, 13.7 ± 6.6 mmHg versus 16.2 ± 6.4 mmHg; *p* < 0.006), but there was no significant difference in IOP reduction between the treatment groups after 3 months [11]. Additionally, 5 years of follow-up revealed that patients with tube shunt placement required additional glaucoma surgery less frequently [12].

The TVT study highlights that tube shunts have the potential to play an increasing role in the surgical management of glaucoma [10]. Moreover, certain groups of patients, such as those with prior intraocular surgery, trauma, or inflammation, are at an increased risk of trabeculectomy failure because of their predisposition to scarring [13, 14]. A glaucoma tube implantation may possibly be more suitable for these patients. Our study aims to report our experience with transscleral suture-fixated PCIOL implantation combined with an AGV implantation.

## 2. Patients and Methods

The retrospective study consisted of 22 consecutive eyes of 22 glaucoma patients who underwent transscleral suture-fixated PCIOL implantation in combination with AGV surgery and had a minimum of 3 months of follow-up. The surgeries were performed by 4 consultant surgeons between 2006 and 2015 at National University Hospital, Singapore. The study adhered to the principles laid out in the Declaration of Helsinki and obtained ethical approval from the National Healthcare Group Domain Specific Review Board (NHG-DSRB).

Among our study patients, the indication for surgery was the presence of both glaucoma and inadequate posterior capsule support, as shown in Table 1.

The data recorded included gender, race, age at surgery, systemic conditions, type of cataracts, type of glaucoma, slit lamp biomicroscopic evaluation, best-corrected visual acuity (BCVA) using Snellen chart, IOP using Goldmann applanation tonometry, number of IOP-lowering medications, intraoperative details, postoperative complications, and reoperation.

Complete success was defined as 6 ≤ IOP ≤ 21 mmHg without IOP-lowering medications at the last follow-up visit, with no reoperation required and no progression to NPL. Failure was defined as IOP > 21 mmHg or <6 mmHg, or if reoperation was required, or if there were IOP-lowering medications at the last follow-up visit, or if there was progression to NPL [15].

### 2.1. Surgical Technique

All patients underwent an AGV insertion, together with transscleral suture-fixated PCIOL (either via anterior segment or posterior vitrectomy). All surgeries were performed at the National University Hospital, Singapore.

Briefly, for AGV surgery, the surgical steps included

- Conjunctival peritomy
- Ahmed tube patency verified
- Plate of seton placed in subtenon space between recti muscles and secured with nylon sutures
- Track of tube into anterior chamber created with 23-gauge needle
- End of tube trimmed to desired length and inserted into the anterior chamber
- Tube secured with nylon sutures to the underlying sclera
- Tutopatch (Regeneration Technologies Inc., Alachua, Florida, United States) glued over path of tube and secured with Tisseel glue (Baxter International Inc., Deerfield, Illinois, United States)
- Conjunctiva closure with nylon sutures

Transscleral suture-fixated PCIOL implantation (anterior segment)

- Intracapsular cataract extraction
- Anterior vitrectomy performed
- Three-piece IOL sutured to sclera with 10/0 prolene

Transscleral suture-fixated PCIOL implantation (posterior vitrectomy)

- Trans-pars plana vitrectomy with lensectomy performed
- Pupil constricted with Miostat and IOL inserted from clear corneal wound
- 10/0 prolene sutures applied to fixate three-piece IOL to sclera

**Figure 1:**
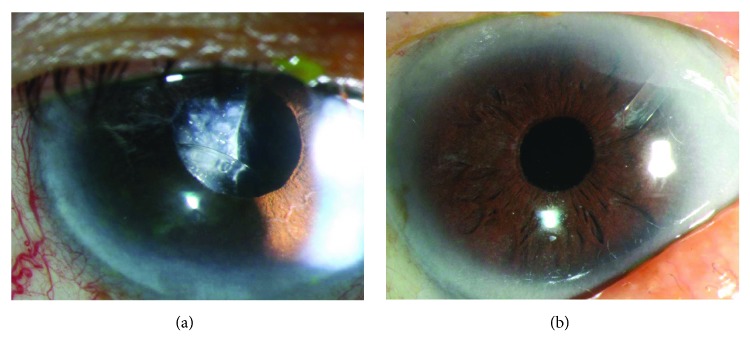
Subluxed intraocular lens (a) and after transscleral suture-fixated posterior chamber intraocular lens and Ahmed glaucoma valve surgery (b).

**Figure 2:**
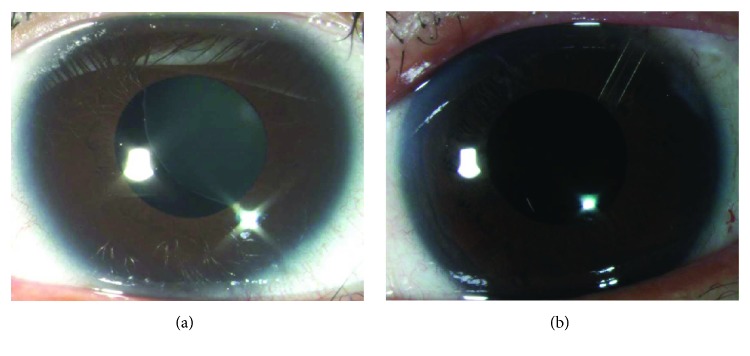
A subluxed crystalline lens (a) and after transscleral suture-fixated posterior chamber intraocular lens and Ahmed glaucoma valve surgery (b).

Figure 1 shows both preoperative and postoperative photos of a patient with a subluxed intraocular lens who underwent transscleral suture-fixated posterior chamber intraocular lens and Ahmed glaucoma valve surgery.  Figure 2 shows preoperative and postoperative photos of a patient with a subluxed crystalline lens who underwent transscleral suture-fixated posterior chamber intraocular lens and Ahmed glaucoma valve surgery.

Upon discharge, patients were prescribed with antibiotic and steroid eye drops, to be used three-hourly to four times daily for the first postoperative week. Antibiotics were stopped one month after the surgery and the steroids were continued with slow taper. In general, all patients were reviewed at approximately one day, one week, one month, three months, and every six months postoperatively.

### 2.2. Statistical Analysis

Statistical analysis was performed using SPSS version 20 (SPSS Inc., Chicago, Illinois, United States). Patient demographics and baseline characteristics were analysed using descriptive statistics; the Wilcoxon signed-rank test was used to compare preoperative and the last visit measurements of IOP, number of IOP-lowering medications, and visual acuity. Visual acuity measured using the Snellen chart was converted to logarithm of minimum angle of resolution (LogMAR) for ease of statistical analysis. A Kaplan-Meier curve was used to assess the probability of success over time. A *p* value < 0.05 was taken to be statistically significant.

## 3. Results

**Figure 3:**
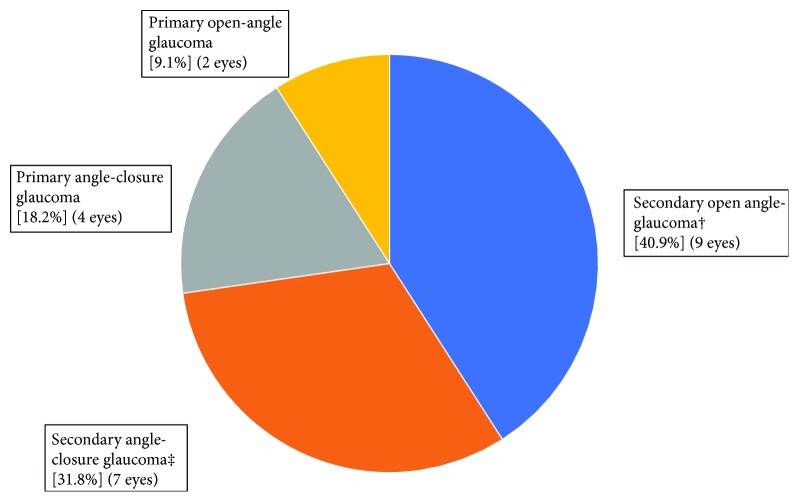
Types of glaucoma in study. † includes angle recession glaucoma (3 eyes), corticosteroid use (2 eyes), uveitis-glaucoma-hyphema syndrome from anterior chamber intraocular lens (1 eye), uveitis (1 eye), postvitrectomy (1 eye), and silicon-oil induced (1 eye). ‡ includes traumatic lens subluxation (3 eyes), lens subluxation of unknown etiology (3 eyes), and neovascular glaucoma (1 eye).

The eyes were followed up for a mean duration of 29.0 ± 19.4 months (median = 27 months), with a range of 3 to 66 months. The overall follow-up rates were 90.6% at 6 months, 77.3% at 12 months, 68.2% at 18 months, and 54.5% at 2 years. Five patients (22.7%) missed one or more follow-up visits, but were not entirely lost to follow-up—they were not excluded from the study. The right eye was operated on in 8 patients (36.4%) and the left eye in 14 patients (63.6%). Nine of the patients were diabetic (40.9%) and 12 of the patients were hypertensives (54.5%).  Table 2 summarizes the study population characteristics and Figure 3 captures the types of glaucoma in this study.

### 3.1. IOP

There was a statistically significant reduction in mean IOP from preoperation to the last follow-up. At the last follow-up, 21 eyes (95.5%) had an IOP ≤ 21 mmHg. Only 1 eye had a pressure of >21 mmHg (22 mmHg). This patient experienced a clot over tube causing tube blockage and vitreous hemorrhage immediately postoperatively, but this was self-limiting without further intervention.

### 3.2. Visual Acuity

The BCVA of the eyes showed a statistically insignificant improvement, from 1.43 ± 1.21 (LogMAR, median = 1.00, range = 0.00–3.20) preoperatively to 1.09 ± 1.31 (median = 0.45, range = 0.00–3.51) at the last visit (*p* = 0.204). At the last follow-up, 3 eyes had a BCVA 6/6 (13.6%). One eye had NPL at the last visit, but preoperatively could only perceive light (PL). It did not experience any complications nor require any further surgical intervention. Six eyes were found to have poorer vision at the last follow-up than preoperatively, but only 3 eyes lost more than 2 lines of vision. Of these 3 eyes, 1 eye experienced retinal detachment, 1 eye experienced tube exposure and endophthalmitis, and 1 eye experienced choroidal detachment, cystoid macular edema, and corneal decompensation.

### 3.3. Number of IOP-Lowering Medications

The mean preoperative number of IOP-lowering medications showed a statistically significant decrease, from 2.45 ± 1.30 (median = 3.00, range = 0–4) to 0.05 ± 0.21 (median = 0.00, range = 0-1) at the last follow-up (*p* < 0.001). Only 1 patient was still taking a single IOP-lowering medication at the last visit. This patient experienced posterior dislocation of the IOL 1 week after the operation and underwent left trans-pars plana vitrectomy, PCIOL explantation, and anterior chamber intraocular lens (ACIOL) implantation 1 week later.

A summary of the outcomes preoperatively and at the last follow-up is captured in Table 3.

#### 3.3.1. Complications and Interventions

Table 4 summarizes the postoperative complications in this study. There were no intraoperative complications. Eight eyes (36.4%) experienced postoperative complications with 4 eyes (18.2%) experiencing multiple complications. However, only 4 of these eyes (18.2%) required reoperation. In terms of postoperative complications, the 3 commonest complications were bullous keratopathy (3 eyes), choroidal detachment (2 eyes), and displacement of PCIOL (2 eyes). Bullous keratopathy tended to occur late (11 months, 22 months, and 29 months after operation), but 2/3 already had preexisting cornea edema prior to their surgery. Choroidal detachment occurred early (1 month and 4 months after operation) and was self-limiting and resolved without operation in 1 patient. PCIOL displacement occurred both late and early (0 month and 23 months)—one of these patients required IOL explantation with trans-pars plana vitrectomy and insertion of ACIOL. The eye that experienced both tube exposure and endophthalmitis underwent corneal graft patch for tube exposure, but later developed hypotony but declined tube explantation. Tube blockage, cystoid macular edema, and vitreous hemorrhage were self-limiting and did not adversely affect BCVA at the last follow-up.

#### 3.3.2. Complete Success

**Figure 4:**
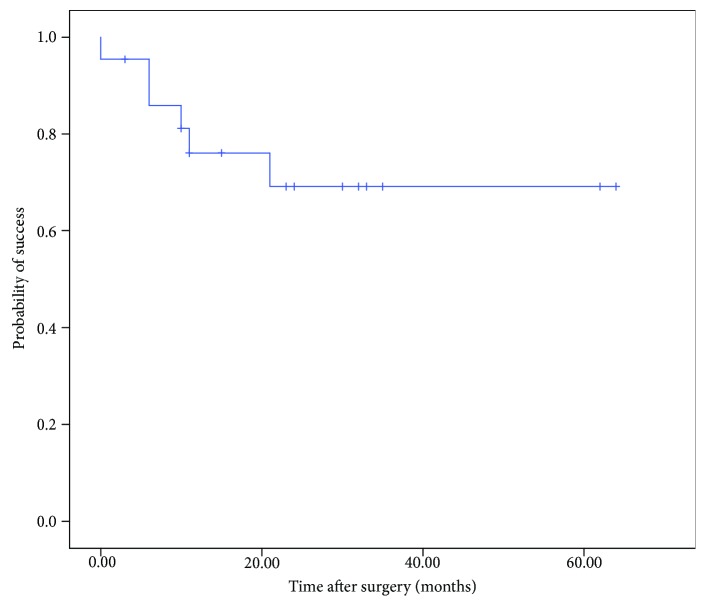
Kaplan-Meier survival plot of probability of failure over time.

Complete success, defined as 6 ≤ IOP ≤ 21 mmHg without medications at the last follow-up visit with no reoperation required and no progression to NPL, was achieved in 16 eyes (72.7%) at the last follow-up. Treatment failure in our study population was due to requirement of reoperation (4 eyes), IOP > 21 mmHg at the last visit (1 eye), IOP < 6 mmHg at the last visit (1 eye), BCVA NPL at the last visit (1 eye), and taking IOP-lowering medication at the last visit (1 eye).  Figure 4 presents a Kaplan-Meier survival plot of probability of failure over time.

## 4. Discussion

This is the first study evaluating the outcomes and complications of transscleral suture-fixated PCIOL combined with Ahmed tube implantation. More than 70% of the patients in our study had secondary glaucoma from previous ocular trauma, surgery, uveitis, and neovascular glaucoma, all of which placed them at increased risk of trabeculectomy failure. We will discuss our findings in relation to the studies of Shin et al. and David et al., all 3 of which are summarized in Table 5. Both Shin et al.'s and David et al.'s studies evaluated the outcomes of transscleral suture-fixated PCIOL combined with trabeculectomy.

Among our study participants, the success rate was 72.7% (16 eyes). The most common reason for failure was requirement of reoperation. Shin et al. had 3 different criteria for success. Their least stringent criteria were as follows: stable visual acuity (improved or within two Snellen lines of preoperative BCVA) and a postoperative IOP of 21 mmHg or less, but with or without the use of medications, 5-fluorouracil (5-FU) needling revision, or incisional glaucoma surgery. Their intermediate criteria included all of the above except that it did not allow for incisional glaucoma surgery, and their most stringent criteria, in addition, did not allow for 5-FU needling revision. Overall, depending on which criteria was applied, 46% to 68% of the patients had both stable visual acuity and satisfactory pressure control at the last postoperative visit [3]. Our criteria for complete success are similar to their most stringent criteria—both have IOP ≤ 21 as a criterion and both do not allow for further surgical intervention. However, our criteria are stricter as we did not allow for the use of medications as well as a postoperative IOP of <6 mmHg, although we did not take stable visual acuity as a prerequisite for complete success.

Our study suggested that our success rates are superior to the rates seen in their study with lower risk of reoperation required. However, this may also have been due to our shorter mean follow-up time (29.0 months versus 38.5 months). Another study done by David et al. evaluated the outcomes of transscleral suture-fixated PCIOL combined with trabeculectomy in patients with subluxated/dislocated lens. In their study, complete success was achieved in 80% of the participants at 2 years follow-up. This is similar to ours, but it must also be noted that their criteria for complete success were more lenient than ours—they allowed for IOP < 6 mmHg, reoperation, as well as progression of vision to NPL.

Among our study participants, transscleral suture-fixated PCIOL combined with Ahmed tube implantation was effective in reducing IOP and reducing number of IOP-lowering medications. This is consistent with the findings of Shin et al. and David et al. In terms of vision, the BCVA of our study's eyes showed that visual acuity did not change significantly after surgery. On the other hand, Shin et al. and David et al. found a statistically significant improvement in BCVA. Although our improvement was statistically insignificant, it could be attributed to the small sample size (22 eyes, as opposed to 56 eyes and 51 eyes). It is also possible that the lack of a significant improvement in vision is due to the fact that the primary indication for surgery in our patients was raised intraocular pressure and not poor preoperative vision.

In terms of the complication profile, our study had 36.4% of patients experienced postoperative complications. In contrast, Shin et al. found that 14% of the eyes experienced postoperative complications, whereas David et al. found that 13.7% of the eyes experienced early postoperative complications and 4% of the eyes experienced late postoperative complications. However, it is worthy to note that most of our complications were self-limiting did not require reoperation and did not adversely affect vision preservation or IOP control in the long run. The complications present in our study were similar to those found by Shin et al. and David et al., such as choroidal detachment, retinal detachment, PCIOL dislocation, and hypotony. Shin et al. found 2 patients with suprachoroidal hemorrhage, which was a complication that did not occur in our study population, but associated with trabeculectomy surgery.

The major limiting factor of our study was its retrospective nature. Therefore, we could not standardize the method and contents of documentation and this gave rise to missing information for some of the study participants. This inconsistency in documentation may also have been due to the fact that the operations were not all performed by a single ophthalmologist. Secondly, due to our small sample size, we could not perform subgroup analysis. Moreover, some statistically insignificant results could possibly become statistically significant with a larger sample size. Finally, because our study comprised almost exclusively of Ahmed tube implantation, the findings may not be applicable to other tubes such as the Baerveldt tube.

## 5. Conclusion

In conclusion, we found that transscleral suture-fixated PCIOL combined with an AGV surgery showed good success rates for IOP control and vision preservation. Although postoperative complications were relatively common, most were self-limiting and did not require reoperation or adversely affect vision preservation or IOP control in the long run. Further studies in other populations that evaluate the outcome of transscleral suture-fixated PCIOL with tube surgery would be helpful, especially if they have a larger sample size. In particular, subgroup analysis would also be important to investigate the predictors of complications and reoperation, which are critical to identifying the group of patients who are most likely to benefit from this procedure.

## Figures and Tables

**Table 1:** Reasons for inadequate posterior capsule support.

	Number of eyes	Percentage (%)
Subluxed cataract	8	36.4
Zonulysis	5	22.7
Subluxed intraocular lens	4	18.2
Complications of anterior chamber intraocular lens	2	9.1
Intraoperative posterior capsular rupture	2	9.1
Aphakia	1	4.5

**Table 2:** Study population characteristics.

Characteristics	*n* (%)
Age (years)	
Mean ± SD	62.4 ± 10.1
Sex	
Male	14 (63.6%)
Female	8 (36.4%)
Ethnicity	
Chinese	18 (81.8%)
Malay	1 (4.5%)
Indian	1 (4.5%)
Others	2 (9.1%)
Follow-up duration (months)	
Mean ± SD	29.0 ± 19.4
Preoperative cup-disc ratio	
Mean ± SD	0.48 ± 0.23
Preoperative visual fields–mean deviation (dB)	
Mean ± SD	−7.64 ± 5.48

**Table 3:** Outcomes preoperatively and at the last follow-up.

	Preoperative	Last follow-up	*p* value^∗^
Intraocular pressure (mmHg)	22.4 ± 6.5	13.9 ± 3.9	<0.001
Best-corrected visual acuity (LogMAR)	1.43 ± 1.21	1.09 ± 1.31	0.204
Number of intraocular pressure-lowering medications	2.45 ± 1.30	0.05 ± 0.21	<0.001

^∗^Wilcoxon signed-rank test.

**Table 4:** Postoperative complications.

Complications	*n* (%)
Bullous keratopathy	3 (13.6%)
Choroidal detachment	2 (9.1%)
Posterior chamber intraocular lens displacement	2 (9.1%)
Endophthalmitis	1 (4.5%)
Tube exposure	1 (4.5%)
Tube blockage by blood over tube	1 (4.5%)
Cystoid macular edema	1 (4.5%)
Vitreous hemorrhage	1 (4.5%)
Retinal detachment	1 (4.5%)

**Table 5:** Study comparison.

	Our study	Shin et al.	David et al.
Success rate (%)^†^	72.6%	46–68%	80%
Intraocular pressure (mmHg) ± SD preoperatively and at the last follow-up	22.4 ± 6.5 to 13.9 ± 3.9 (*p* < 0.001)	22.9 ± 10.9 to 16.7 ± 6.7 (*p* = 0.0005)	26.3 ± 11.5 to 13 ± 4.6 (*p* < 0.001)
Best-corrected visual acuity (LogMAR) ± SD preoperatively and at the last follow-up	1.43 ± 1.21 to 1.09 ± 1.31 (*p* = 0.204)	—	2.9 ± 0.8 to 0.3 ± 0.7 (*p* < 0.001)
Number of intraocular pressure-lowering medications ± SD preoperatively and at the last follow-up	2.45 ± 1.30 to 0.05 ± 0.21 (*p* < 0.001)	2.3 ± 0.9 to 1.9 ± 0.9 (*p* = 0.0002)	1.4 ± 0.8 to 0.5 ± 0.7 (*p* < 0.001)
Complication rate (%)^‡^	9.1% (early complications) 27.3% (late complications)	14%	13.7% (early complications) 4% (late complications)
Percentage of eyes requiring reoperation (%)	18.2%	33.9%	—

^†^Different definition of success rates. ^‡^Early complications are defined as complications occurring within 3 months of operation, whereas late complications are defined as complications occurring at least 3 months after the operation.
